# 2365. Effectiveness of COVID-19 Vaccines Against Medically Attended COVID-19 in Pregnant Persons Within the VISION Network, December 2021 - April 2023

**DOI:** 10.1093/ofid/ofad500.1986

**Published:** 2023-11-27

**Authors:** Allison Avrich Ciesla, Ruth Link-Gelles, Katherine E Fleming-Dutra, Victoria Lazariu, Kristin K Dascomb, Shaun J Grannis, Toan Ong, Peter J Embi, Sarah W Ball, Manjusha Gaglani, Anupam Kharbanda, Karthik Natarajan, Ousseny Zerbo, Gabriela Vazquez-Benitez, Stephanie Irving

**Affiliations:** Centers for Disease Control and Prevention, Rochester, New York; Centers for Disease Control and Prevention, Rochester, New York; Centers for Disease Control and Prevention, Rochester, New York; WESTAT, Rockville, Maryland; Intermountain Healthcare, Murray, Utah; Indiana University, Indianapolis, Indiana; University of Colorado Anschutz Medical Campus, Centennial, Colorado; Vanderbilt University Medical Center, Nashville, Tennessee; Westat, Newton, Massachusetts; Baylor Scott & White Health, Temple, TX; Children's Minnesota Research Institute, Minneapolis, MN; Columbia University, New York, New York; Division of Research Kaiser Permanente Vaccine Study Center, Oakland, California; HealthPartners Institute, bloomington, Minnesota; Kaiser Permanente Center for Health Research, Portland, Oregon

## Abstract

**Background:**

Pregnant people have an increased risk of severe COVID-19, including hospitalization and critical illness. Currently, pregnant people are recommended to receive the same vaccinations as non-pregnant people of the same age and underlying health status (i.e., with or without immunocompromising conditions); however, additional data are needed to inform policy decisions around the potential need for an extra dose during pregnancy to protect mother and infant. Our goal was to estimate effectiveness of COVID-19 vaccination against medically attended COVID-19 among pregnant people, during predominance of the Omicron variant.

**Methods:**

The VISION Network conducted a test-negative, case-control study including emergency department/urgent care (ED/UC) encounters December 2021–April 2023 among immunocompetent pregnant people between the ages of 18-45 years. Encounters were included if COVID-19-like illness (CLI) was documented, and the pregnant person underwent SARS-CoV-2 testing within 14 days prior to the encounter. We compared the odds of vaccination among pregnant persons who tested positive with the odds of vaccination among those who tested negative. Monovalent vaccine effectiveness (VE) was calculated as 1-adjusted odds ratio multiplied by 100.

**Results:**

Among 10,631 eligible CLI-associated ED/UC encounters, 2,022 (19%) were SARS-CoV-2 positive (Table). Of these, 52% of cases and 44% of controls were unvaccinated and 14% of cases and 23% of controls had received a primary series with at least 1 booster. VE of a complete monovalent primary series was 19% (95% CI: 8-29%); VE of a primary series plus a monovalent booster was 39% (95% CI: 27-50%). Median time since last dose was 349 and 219 days, respectively (Figure). VE of bivalent doses and time since last dose within the VISION Network will be available in the coming months.

**Figure:**
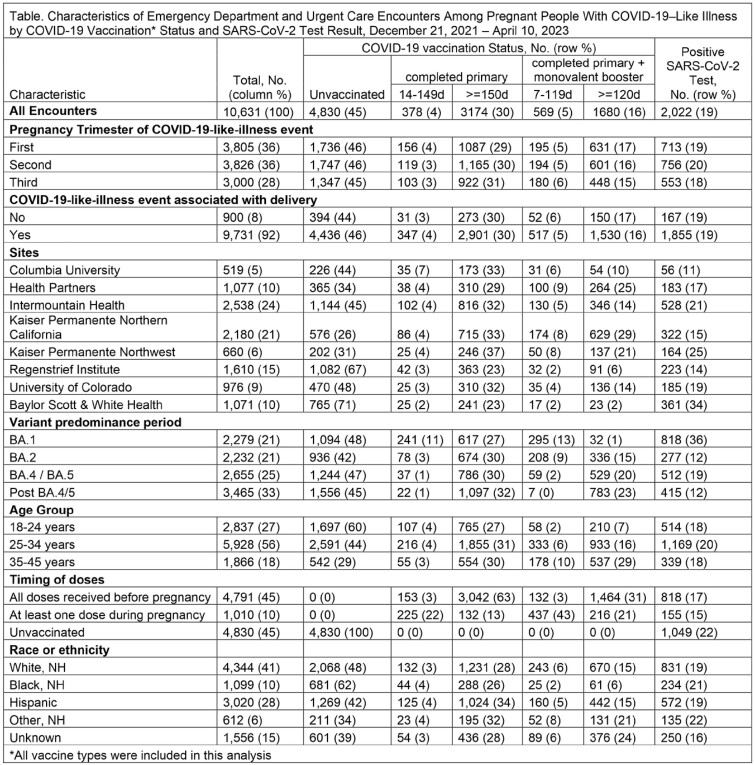

**Figure:**
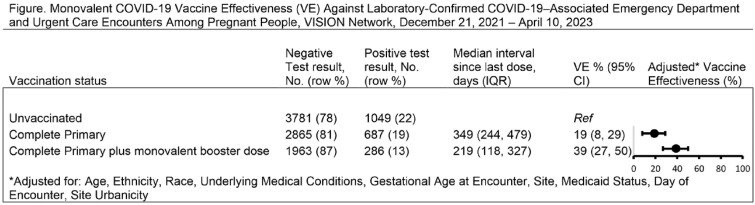

**Conclusion:**

Monovalent COVID-19 vaccines helped provide protection against medically attended COVID-19 among pregnant people. Pregnant people should stay up to date with all recommended vaccinations.

**Disclosures:**

**Gabriela Vazquez-Benitez, PhD, MSc**, Abbvie: Grant/Research Support|Sanofi Pasteur: Grant/Research Support

